# Validation of vessel size imaging (VSI) in high-grade human gliomas using magnetic resonance imaging, image-guided biopsies, and quantitative immunohistochemistry

**DOI:** 10.1038/s41598-018-37564-w

**Published:** 2019-02-26

**Authors:** Ararat Chakhoyan, Jingwen Yao, Kevin Leu, Whitney B. Pope, Noriko Salamon, William Yong, Albert Lai, Phioanh L. Nghiemphu, Richard G. Everson, Robert M. Prins, Linda M. Liau, David A. Nathanson, Timothy F. Cloughesy, Benjamin M. Ellingson

**Affiliations:** 1 UCLA Brain Tumor Imaging Laboratory (BTIL), Center for Computer Vision and Imaging Biomarkers, David Geffen School of Medicine, University of California Los Angeles, Los Angeles, CA USA; 2 Department of Radiological Sciences, David Geffen School of Medicine, University of California Los Angeles, Los Angeles, CA USA; 3 Department of Bioengineering, Henry Samueli School of Engineering and Applied Science, University of California Los Angeles, Los Angeles, CA USA; 4 Division of Neuropathology, Department of Pathology and Laboratory Medicine, David Geffen School of Medicine, University of California Los Angeles, Los Angeles, CA USA; 5 Department of Neurology, Ronald Reagan UCLA Medical Center, University of California Los Angeles, Los Angeles, CA USA; 6 Department of Neurosurgery, Ronald Reagan UCLA Medical Center, University of California Los Angeles, Los Angeles, CA USA; 7 Department of Molecular and Medical Pharmacology, David Geffen UCLA School of Medicine, Los Angeles, CA USA; 8 UCLA Neuro Oncology Program, David Geffen School of Medicine, University of California Los Angeles, Los Angeles, CA USA

## Abstract

To evaluate the association between a vessel size index (VSI_MRI_) derived from dynamic susceptibility contrast (DSC) perfusion imaging using a custom spin-and-gradient echo echoplanar imaging (SAGE-EPI) sequence and quantitative estimates of vessel morphometry based on immunohistochemistry from image-guided biopsy samples. The current study evaluated both relative cerebral blood volume (rCBV) and VSI_MRI_ in eleven patients with high-grade glioma (7 WHO grade III and 4 WHO grade IV). Following 26 MRI-guided glioma biopsies in these 11 patients, we evaluated tissue morphometry, including vessel density and average radius, using an automated procedure based on the endothelial cell marker CD31 to highlight tumor vasculature. Measures of rCBV and VSI_MRI_ were then compared to histological measures. We demonstrate good agreement between VSI measured by MRI and histology; VSI_MRI_ = 13.67 μm and VSI_Histology_ = 12.60 μm, with slight overestimation of VSI_MRI_ in grade III patients compared to histology. rCBV showed a moderate but significant correlation with vessel density (r = 0.42, p = 0.03), and a correlation was also observed between VSI_MRI_ and VSI_Histology_ (r = 0.49, p = 0.01). The current study supports the hypothesis that vessel size measures using MRI accurately reflect vessel caliber within high-grade gliomas, while traditional measures of rCBV are correlated with vessel density and not vessel caliber.

## Introduction

Neovascularization in gliomas plays an important role in response to anti-tumor strategies^1,2^. Various signaling pathways regulate this complex mechanism, including vascular co-option, angiogenesis, vascular mimicry, and endothelial cell trans-differentiation^3,4^. It is known that this tumor vasculature is functionally and morphologically abnormal^5^. The quantification of abnormal vessels on histology is a standard indicator of poor outcome for glioma patients^6–8^. Using magnetic resonance imaging (MRI), several methods have been proposed that yield physiologic information about tumor vasculature, including blood flow and volume^9,10^. Theoretical Monte-Carlo simulations have suggested that T_2_- and T_2_*-weighted images acquired during dynamic susceptibility contrast (DSC) perfusion imaging are sensitive to microvasculature and larger vessels, respectively^11,12^, by exploiting the differences in transverse relaxation rates R_2_* and R_2_ during the passage of contrast bolus through the vasculature^13^. This effect observed in small vessels is related to the magnitude of water diffusion, which is equivalent to the local susceptibility gradient. When the vascular bed is quantified with spin-echo (SE) sequence, the blood flow and volume are capillary weighted with a radius lower than 10 μm^13^, while with gradient-echo (GE) sequence, the hemodynamic parameters are weighted to total vessels of all size^14^. In this way, separate or simultaneous acquisitions of SE and GE parameters have been speculated to be useful in more functional information of blood vessels architecture and its oxygenation^15–18^. For example, the ratio of peak ΔR_2_*/ΔR_2_ has been shown to correlate with histologically derived measures of vessel size in a preclinical C6 glioma xenograft model^14^. This promising preliminary evidence suggesting a close link between the average vessel size index derived from MRI (VSI_MRI_) and histology (VSI_Histology_) using preclinical models^16,19^.

A separate model for estimating vessel size was established by Kiselev *et al*.^17,20^ and incorporates measures of both cerebral blood volume and water diffusivity. In this model, the proportionality constant to scale differences between MRI and histology has not been thoroughly validated^17^. Additionally, in the previous publications, the normalized rCBV was set generically to 6%, which represents an extreme physiological conditions^21^. Recently, a single study involving glioma patients confirmed the association between this model and vascular morphometry using CD34 endothelial cell marker expression^22^; however, despite the high number of patients and biopsy samples in each individual patient, this study reported very few identifiable vessels (~10 per sample) in some glioma patients, likely related to the small biopsy size (1–1.5 mm in diameter).

In this current work, we quantified relative cerebral blood volume (rCBV) and VSI_MRI_ using a spin-and-gradient echo echoplanar imaging (SAGE-EPI) sequence during DSC perfusion MRI in patients with high-grade gliomas. The correlation between VSI_MRI_ and rCBV with vessel density and caliber from image-guided biopsies were examined after staining with endothelial cell marker, CD31, chosen as a specific marker of undifferentiated and differentiated microvasculature^23,24^. We hypothesized that Kiselev’s model, which provide several corrections (both diffusion and perfusion components) to estimate vessel size, would be strongly associated with VSI_Histology_.

## Results

**Figure 1:**
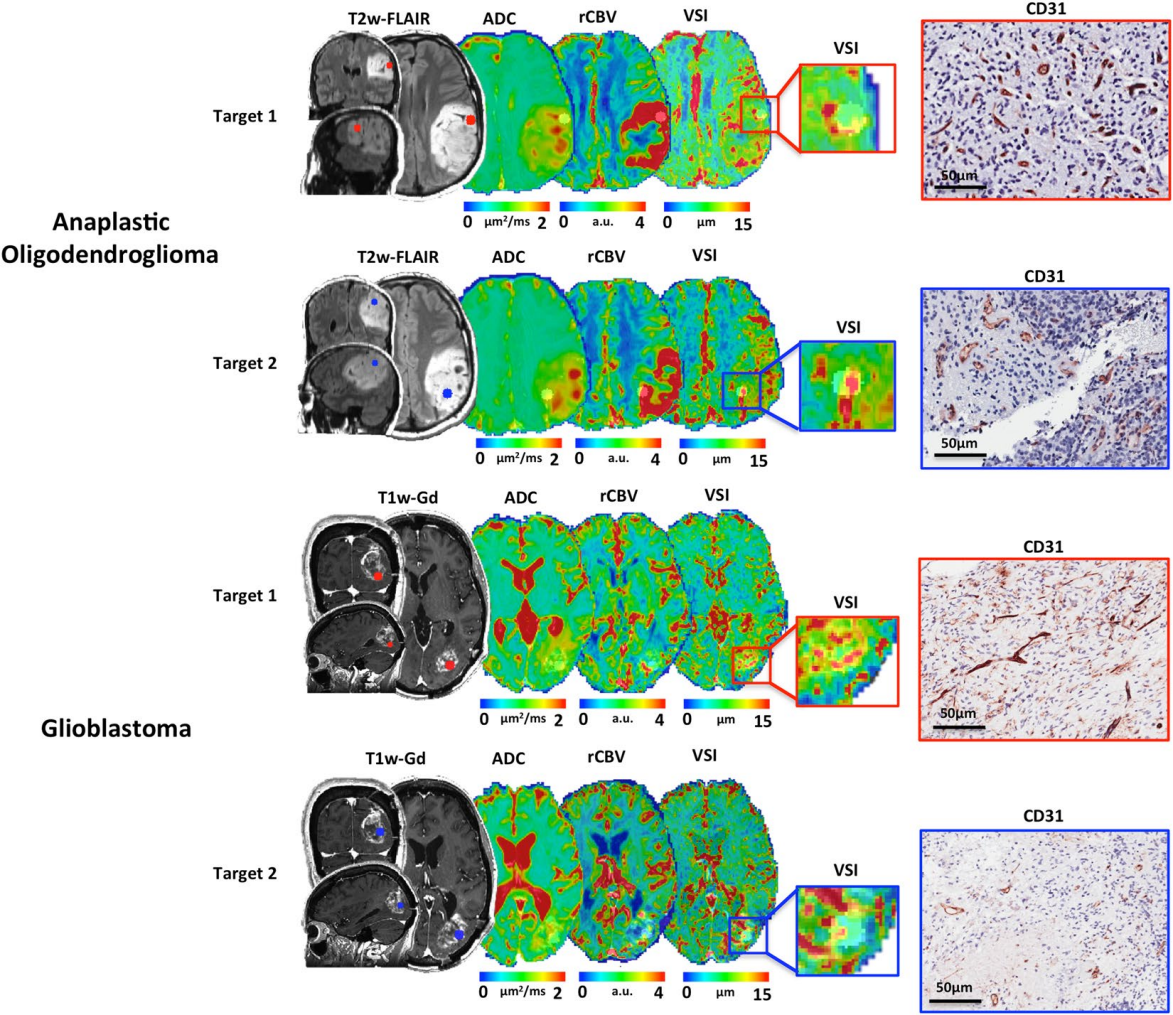
Two representative high-grade glioma patients are reported with their respective biopsy targets. T2w-FLAIR (upper) or post-contrast T1w (bottom) images were used for anatomical reference in tissue sampling in various tumor grades. ADC, rCBV and VSI_MRI_ maps are shown for each patient. The first patient (upper) has a WHO grade III, Anaplastic Oligodendroglioma. The brain lesion is located in the left frontal lobe. The second patient (bottom) is a WHO Grade IV glioblastoma with a brain lesion located in the left parietal lobe. An elevated ADC and rCBV showed in both patients within tumor areas. Glioblastoma patient show an increased heterogeneity of vessel size within and around enhancing areas. Examples of tissue slides with stained CD31 positive vessels for each representative target. As observed with VSI_MRI_, in both grade III and IV, CD31 positive vessels are present with spatial heterogeneity in terms of density and morphometry.

Typical example of T2w-FLAIR, post-contrast T1w, ADC, rCBV, VSI images as well as CD31 staining of high-grade glioma patients are shown in Fig. 1. The first patient, bearing an anaplastic oligodendroglioma lesion in the left frontal lobe, illustrated a lesion with hyperintensities on T2w-FLAIR images, increased ADC and rCBV as compared to corresponding NAWM (Fig. 1, upper). In contrast, VSI_MRI_ maps showed potential hyper-dense and large vessels in the edges of the lesion as demonstrated with targets overlaid on VSI maps (rectangles) and confirmed with CD31 staining. Patient B, harbored a glioblastoma in left parietal lobe (Fig. 1, bottom). This patient has a heterogeneous ring-enhancing lesion with perilesional edema, elevated ADC within the area of edema, increased rCBV in the posterior part of the tumor with increased and spatially heterogeneous measures of VSI_MRI_. As confirmed with MRI and histology, high-grade gliomas patients demonstrate intra-tumoral vascular heterogeneity (CD31 staining) as highlighted by different targets from each patient.

We next assessed for each biopsy region, histological and MRI-derived features. From three-dimensional, 5 mm radius biopsy targets, we found a median number of vessel of 90 [interquartile range = 64.6–117.7], a median density of 40.6 per mm^2^ [interquartile range = 29.8–78.6 per mm^2^] and a median vessel diameter of 12.6 [interquartile range = 10.5–14.1 μm]. A median ADC of 1.30 μm^2^/ms [interquartile range = 1.05–1.63 μm^2^/ms] was measured within 5 mm biopsy targets, with a median rCBV of 1.39 [interquartile range = 1.00–2.36] and a median VSI of 13.6 μm^2^ [interquartile range = 11.98–15.06 μm^2^/ms].

**Figure 2:**
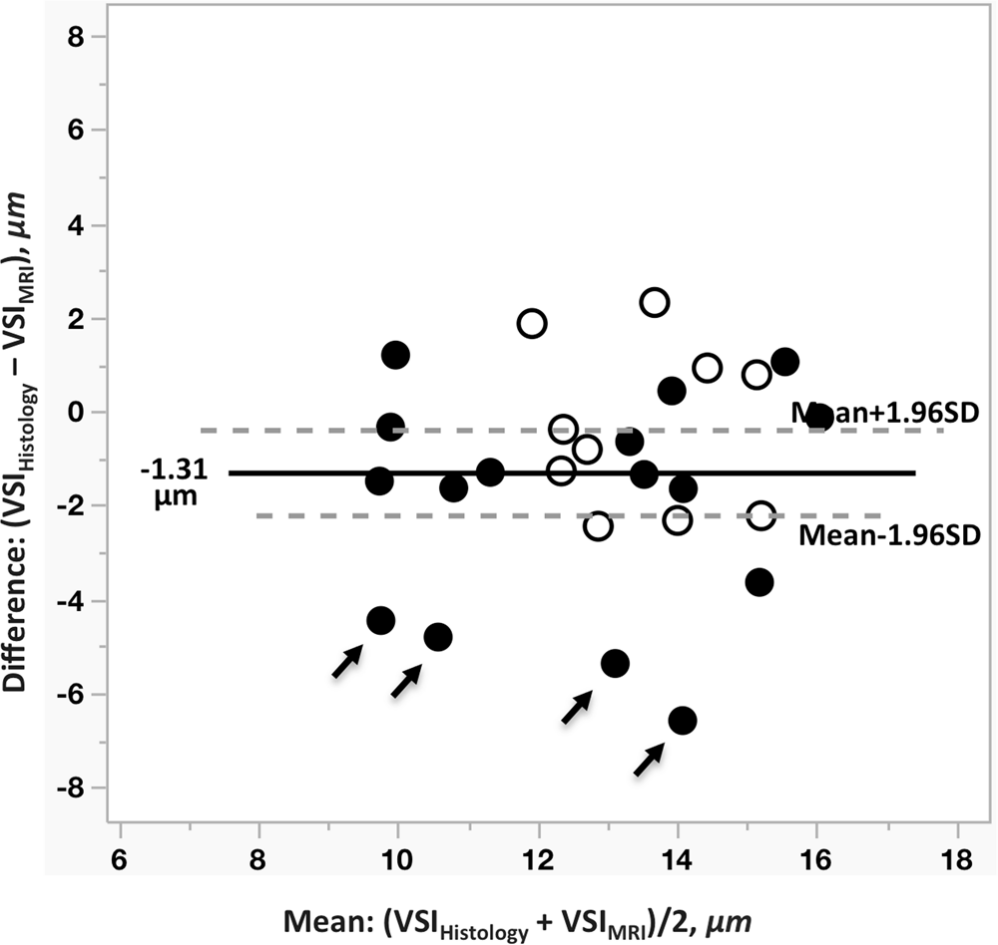
Bland-Altman plot represents potential bias of agreement between these two techniques. A number of points display higher difference between VSI_MRI_ and VSI_Histology_, especially in grade III (black arrows). Filled and unfilled circles presenting targets from grade III and grade IV, respectively.

Bland-Altman plot (Fig. 2A) suggests a significant difference between VSI measures between MRI and histology. Mean difference between MRI and histology was −1.13 μm (p = 0.03, Student’s t-test) with 1.96 standard deviation values of the differences ranged from −0.41 μm to −2.22 μm for respectively, upper and lower limits. Black arrows in Fig. 2 indicate biopsy targets in WHO III gliomas where VSI_MRI_ overestimated vessel caliber compared with histology.

**Figure 3:**
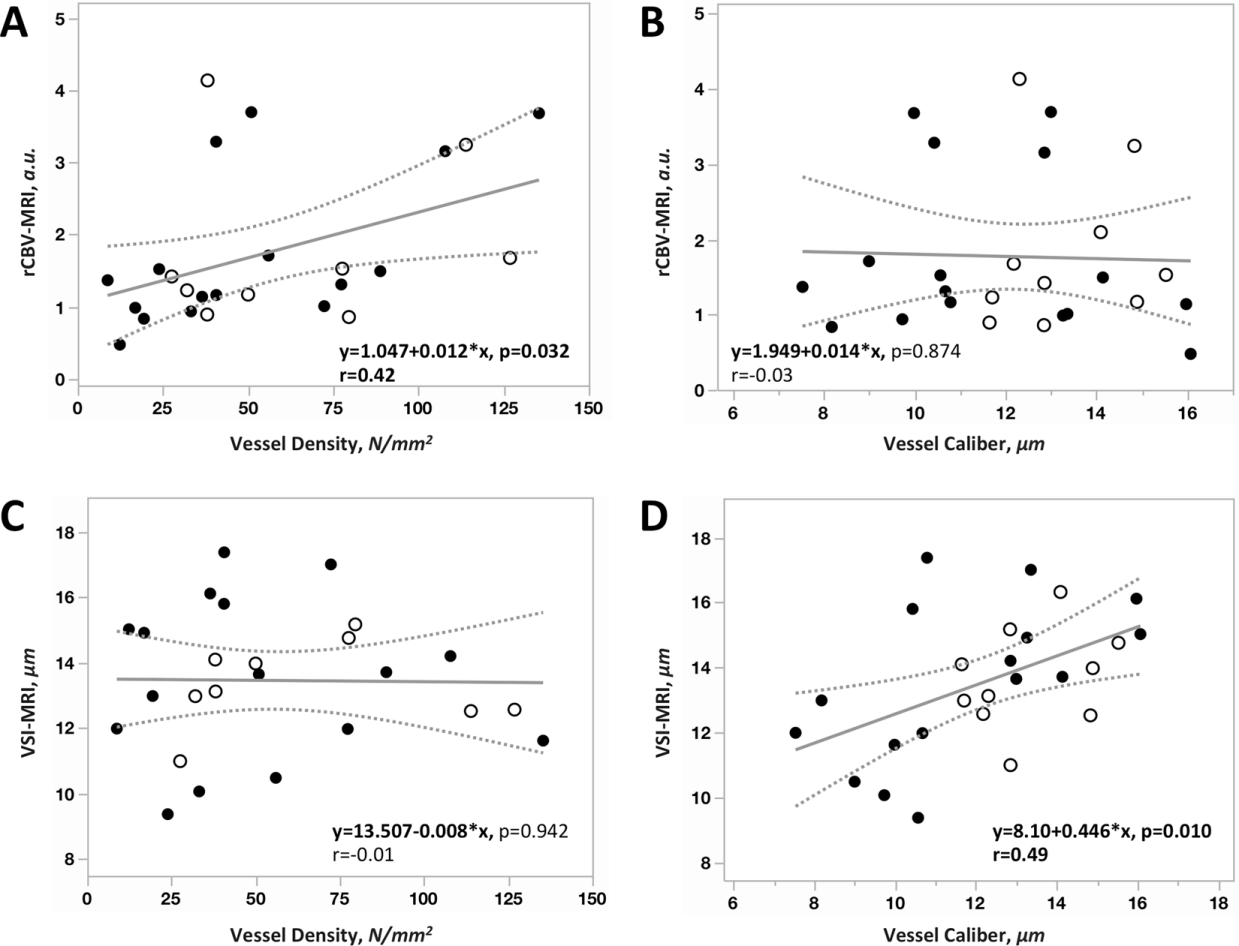
Linear correlation was performed between rCBV, VSI_MRI_ and their corresponding histology value; vessel density and vessel caliber (VSI_Histology_). Positive linear correlation was found between rCBV vs. vessel density (**A**, r = 0.42, p = 0.032) as well as between VSI_MRI_ and vessel caliber (**D**, r = 0.49, p = 0.010). However, no direct relationship was observed between rCBV and vessel caliber (**B**). VSI_MRI_ measures are independent of vessel density (**C**). The filled and unfilled circles presenting targets from grade III and grade IV, respectively.

When directly comparing MR-based perfusion measures with histology (Fig. 3A–D), MRI-derived rCBV showed a moderate, but statistically significant correlation with histology-derived estimates of vessel density (r = 0.42, p = 0.032, Fig. 3A), but not with vessel caliber (r = −0.03, p = 0.874, Fig. 3B). Consistent with our original hypotheses, VSI_MRI_ was significantly correlated with vessel caliber (r = 0.49, p = 0.010, Fig. 3D), but was not correlated with vessel density obtained from histology (r = −0.01, p = 0.942, Fig. 3C).

## Discussion

The current study supports the hypothesis that VSI measured using MRI is highly correlated with the actual vessel caliber in underlying tumor tissue within high-grade gliomas. Additionally, the current study also supports the notion that rCBV measures from MRI correlated with vessel density within the tumor.

There have been several MRI based studies demonstrated the feasibility of VSI or similar measurements. While early studies by Donahue *et al*.^25^ and Schmainda *et al*.^26^ have demonstrated the clinical feasibility of measures of mean vessel diameter (mVD), VSI measures that take into account both rCBV and ADC, which can influence vessel size measurements, have only been thoroughly evaluated orthotopic brain tumor models, which found with good quantitative agreement of histology^19,27^. However, all these studies have used high field MRI (up to 4.7 T) with potential imperfections of main B_0_ and transmit B_1_ magnetic fields, which should be considered^28^ for the appropriate quantification ΔR_2_*, ΔR_2_ and finally VSI. A recent publication using Tropès model showed that, with high-field MRI (7 T), VSI measurements tend to overestimate as compared to two-photon laser scanning microscopy^29^. This observation may be due to underestimation of blood volume in the tumor and/or heterogeneous distribution of vessel radii.

Recently, the Kiselev model VSI_MRI_ has been reported with adjusted rCBV values for healthy tissue to more typical values for blood volume (3.2%)^22^. The former study also compared histological findings (vessel diameter) with MRI results (rCBV and VSI). With an averaged rCBV of 5.99% (uncorrected for contrast leakage); an ADC of 1.02 μm/ms, authors reported an average VSI of 67.13 μm in high-grade glioma, which is two times higher than what they reported for mean vessel diameter (31 μm) but has good agreement with maximum vessel diameter (69.8 μm). However, the tissue targeting protocol of that study may be biased, as for 4 glioblastoma patients, authors reported less than 6 detected vessels with CD34 endothelial marker. This marker is known to be specific for differentiated, well-formed vessels (including normal vessels)^30^ which makes results difficult to interpret.

With the same range of healthy tissue blood volume, 3%, Xu *et al*.^31^, reported an average VSI of 13.8 μm in gray matter and 13.1 μm in white matter. These results of healthy tissues are correlates well with vessel radius values reported by Christen *et al*.^30^, (12.6 ± 2.4 μm with 3.1% of CBV in gray matter). Note that in our study, the rCBV is only normalized to contralateral NAWM and the average rCBV was 0.96, which in turns, results to an average VSI_MRI_ of normal appearing brain 4.51 μm (results not shown). Additionally, a study of confocal laser microscopy reports an average vessel diameter of human cortex lower than MRI approaches; 7.82 ± 3.52 μm^31^, which is in accord to other mammalian neocortex vascular diameter (e.g. cat and rat; ranged from 4.2 to 7 μm), reviewed by Pawlik *et al*.^32^. Indeed, the fact that VSI_MRI_ correlates to VSI_Histology_ independently of vessel density is very promising; however, some overestimation is reported in our current study, especially in the WHO grade III patients and in preclinical studies^15,16^. This overestimation from VSI_MRI_ is consistent with results of Kellner *et al*.^22^, as well as from a rodent study^19^, and occurs especially in small vessel sizes. The following imperfection could be overcome by assessing VSI_MRI_ with more flexible topological models including vessel length, radius, and vessel orientation angles^33^. Additionally, our observations regarding the association between rCBV and vessel density appear consistent with previously reported results from tumor bulk^34^; however, tumor vessel size heterogeneity often influences the reliability of rCBV estimates in comparison with histology^35^.

There were certain limitations of our experimental setup that should be addressed. First, the limited spatial resolution and the registration of lower-resolution SAGE-EPI to high-resolution 3D-T1w images may have resulted in potential bias. It is important to note that precise targeting of brain tissue during biopsy is a significant technical challenge, as inherent changes in the brain position during craniotomy may occur, which could directly affect accurate MRI-guided sampling of tumor specimens. Additionally, the use of rCBV as a normalized approximation of rCBV is another limitation of VSI modeling which could have led to inaccuracies. Also, the specificity of VSI_MRI_ to perfused vessels can also result in discordance between VSI measurements and histology. Moreover, the “delta” term that describes the residual signal differences from imperfectly matched slice profiles may vary over time and may be dependent on the radiofrequency architecture and other details. However, this did not vary more than 3–6% across patients over time in our study (results not shown), so it likely did not influence our results. Finally, contrast-to-noise (CNR) ratio over time may have been affected by use of a single dose of contrast agent, particularly for estimation of R_2_, so this should also be recognized.

In summary, the current study estimated VSI with SAGE-EPI in high-grade glioma patients and correlated this measurement with histological characteristics of the vessel architecture. Results demonstrated that VSI measured with MRI is correlated with vascular caliber, while vessel density is mostly linked to measures of rCBV.

## Material and Methods

### Patients

This study was performed in accordance with the Health Insurance Portability and Accountability Act (HIPAA), and all patients provided signed, informed written consent for all experimental protocols used in the current, institutional review board approved study (UCLA Medical IRB 2, #14-001261). Eleven patients with histologically confirmed high-grade glioma (7 WHO grade III [4 *de novo* and 3 at first recurrence] and 4 WHO grade IV glioblastoma [2 *de novo* and 2 at first recurrence]) have been enrolled in this retrospective study. From 4 *de novo* grade III patients, 3 were anaplastic oligodendroglioma and one diffuse astrocytoma (IDH mutant). From remaining 3 recurrent grade III patients, 2 were anaplastic gangliogliomas and one anaplastic astrocytoma. For 5 enrolled recurrent patients, 4 received standard craniotomy, followed by chemoradiation prior to the second craniotomy. One anaplastic oligodendroglioma received only craniotomy in 2009 and image-guided biopsy was performed in 2015. Of the 11 enrolled patients (9 men and 2 woman), the median age was 50.8 years ranged from 28.5 to 67.9 years. Both MRI and neuropathology specimens were obtained and analyzed with respect to local ethical committee approval. In total, 26 MRI-based targets were biopsied and analyzed.

### Magnetic Resonance Imaging

All MRI images were acquired using a 3 Tesla MRI system (Siemens; Erlangen, Germany) in compliance with the international standardized brain tumor imaging protocol (BTIP)^36^. Briefly, 1 mm isotropic, 3D MPRAGE T1-weighted images were acquired prior to contrast injection, along with axial T2-weighted images and T2-weighted fluid attenuation inversion recovery (FLAIR) images. Axial diffusion-weighted imaging (DWI) was performed using a single-shot echo-planar imaging with three b values (0, 500 and 1000 sec/mm^2^) to compute the apparent diffusion coefficient (ADC). T_2_-, T_2_-weighted FLAIR, and DWI were all collected with 3-mm slice thickness and no interslice gap.

VSI_MRI_ and rCBV were calculated by acquiring dynamic SAGE-EPI data during contrast injection. A pre-dose 0.025 mmol/kg of Gd-DTPA was first administrated reduce contrast extravasation, followed by a bolus dose of 0.075 mmol/kg. The SAGE-EPI readout consisted of two gradient echoes (TE_1_ = 14.0 ms; TE_2_ = 34.1 ms), an asymmetric spin echo (TE_3_ = 58.0 ms) and a spin echo (TE_4_ = 92.4 ms) EPI train with GRAPPA acceleration factor of 3. The repetition time was 2000 ms with a slice thickness of 5 mm and no additional spacing between slices. The resolution was set to 1.875 × 1.875 mm with a total matrix size of 240 × 218 mm. A total of 90 repetitions were obtained over 19 axial slices. Following DSC perfusion acquisition, a parameter matched, 1-mm isotropic, post-contrast 3D MPRAGE T1-weighted dataset was acquired according to BTIP.

### MRI post-processing

Dynamic susceptibility contrast based relative cerebral blood volume (rCBV) maps were calculated using an in-house bi-directional contrast agent leakage correction algorithm that accounts for both contrast flux out of and into the vasculature^37^. Normalization of rCBV maps was performed by comparison to contralateral normal appearing white matter (NAWM).

Estimation of VSI_MRI_ was based on the Kiselev model^17^, which is built upon on the basis of Tropès model^15^ with additional consideration for rCBV values and use the ratio of ΔR_2_*/ΔR_2_ as a result of average vessel size index, expressed in μm:

1 $${{\rm{VSI}}}_{{\rm{\mu }}{\rm{m}}}=0.867{({\rm{rCBV}}\cdot {\rm{ADC}})}^{1/2}(\frac{{\rm{\Delta }}{{\bf{R}}}_{2}^{\ast }}{{{\rm{\Delta }}{\rm{R}}}_{2}^{3/2}})$$

where $\Delta {R}_{2}^{\ast }$ and $\Delta {R}_{2}$ represents the maximum changes in the transverse relaxation rates obtained from solving the following linear equation^38^:

2 $${\rm{A}}={{\rm{Y}}}^{-{\rm{1}}}{\rm{S}}$$

where

3 $${\rm{S}}=(\begin{array}{c}\mathrm{ln}({{\rm{S}}}_{1})\\ \mathrm{ln}({{\rm{S}}}_{2})\\ \mathrm{ln}({{\rm{S}}}_{3})\\ \mathrm{ln}({{\rm{S}}}_{4})\end{array}),{\bf{Y}}=(\begin{array}{cccc}1 & 0 & -{{\rm{TE}}}_{1} & 0\\ 1 & 0 & -{{\rm{TE}}}_{2} & 0\\ 1 & -1 & -{{\rm{TE}}}_{4}+{{\rm{TE}}}_{3} & {{\rm{TE}}}_{4}-2\cdot {{\rm{TE}}}_{3}\\ 1 & -1 & 0 & -{{\rm{TE}}}_{4}\end{array}),{\bf{A}}=(\begin{array}{c}\mathrm{ln}({{\rm{S}}}_{0})\\ \mathrm{ln}({\rm{\delta }})\\ {{\rm{R}}}_{2}^{\ast }\\ {{\rm{R}}}_{2}\end{array})$$

where S_n_ is signal magnitude for the n^th^ echo and δ is the differences in residual signal differences introduced from imperfectly matched slice profiles. Those discordances are related to the echo trains, before and after refocusing pulse^39^.

### Image registration

All images (T2w, FLAIR, ADC, rCBV, and VSI_MRI_) were registered to 1-mm isotropic post-contrast T1-weighted images using a 12-degree-of-freedom, automated linear registration tool using a correlation ratio cost function (FSL-FLIRT, http://www.fmrib.ox.ac.uk/fsl/). All registered maps were visually inspected and, if necessary, manually corrected in the event of misregistration.

### Image-guided biopsy and Immunohistochemistry

After image acquisition, one to three (5 mm radius) targets were identified on post-contrast T1w and/or fused T2w-FLAIR images within contrast-enhancing (tumor core) and non-enhancing tumor regions, respectively. These targets were loaded into BrainLab Neuronavigation software (BrainLab AG, Munich, Germany). Following target identification, a critical review was performed by the primary neurosurgeon to make sure that targets were within the final resection volume, did not affect brain eloquent areas (assessed by blood level dependent contrast (BOLD) activation maps) and were not within the main trajectory of large white matter tracts (assessed by diffusion tensor imaging).

Following image-guided resection, biopsy samples were transferred to the Department of Pathology & Laboratory Medicine for immunohistochemistry (IHC) staining. IHC using an antibody against the endothelial cell marker CD31 has been chosen. Staining was performed on 4 μm paraffin-embedded sections after initial dewaxing with xylene and rehydration through graded ethanol, followed by antigen retrieval with a pH 6.0 Antigen Retrival Solution (Biocare Medical) in a Decloaking pressure cooker at 95 °C for 40 min. Tissue sections were then treated with 3% hydrogen peroxide (LOT 161509; Fisher Chemical) and with Background Sniper (Biocare Medical, Concord, CA, USA) to reduce nonspecific background staining. All slides were then incubated at room temperature for 80 min with ready to use primary antibody for CD31 (Biocare, 090215) followed by detection with the MACH 4 Mouse HRP- Polymer Detection kit (Biocare Medical). VECTOR NovaRED (SK-4800; Vector Laboratories, Inc.) was applied as chromogen.

### Segmentation of blood vessels from CD31 staining and quantification of VSI_Histology_

Segmentation of CD31 was performed on 2D stained slices based on the CAIMAN algorithm^40^. Briefly, the algorithm exploits the distinctive hues of stained vascular endothelial cells, cell nuclei and background. A region-growing algorithm using the seeds created with the previous step and a 3D Hue, Saturation, Value (HSV) color model. Three major morphological tasks were then performed: (1) joining separate objects that were likely to belong to a single vessel; (2) closing objects that had a narrow gap around their periphery; and (3) splitting objects with multiple lumens into individual vessels. A hole fill was performed to include vessel lumen in the calculation of vessel radius. Manual correction was performed on the stained slices with obvious errors. A total of 3 regions of interest were selected for each target and the average quantification of these 3 values was attributed to each target.

After segmentation, vessel density (number divided by the total area of the sample) was computed and expressed in *N*/*mm*^2^. Quantification of VSI_Histology_ on the stained slides were then performed following a previously described model^16^ (assuming vessels are randomly oriented, continuous cylinders with different radii):

4 $$VS{I}_{histo}={(\frac{{\sum }_{i}n({r}_{i}).{r}_{i}^{4/3}}{{\sum }_{i}n({r}_{i}).{r}_{i}^{2}})}^{-3/2}$$

where $n({r}_{i})$ is the number of vessels with radius $r$. Final results are expressed in μm.

### Statistical analyses

A nonparametric Wilcoxon-Mann-Whitney test was used to assess potential statistical differences in both WHO grade III and IV for all MRI and histology-derived parameters. Regression was performed to assess the degree of agreement between MRI (rCBV and VSI_MRI_) and histology (density and VSI_Histology_). An alternative analysis of potential bias determination between these two techniques (VSI_MRI_ vs. VSI_Histology_) was performed using a Bland-Altman test. The difference between VSI_MRI_ vs. VSI_Histology_ was assessed using Student’s *t*-test, after checking the normality of each distribution using a Shapiro-Wilk test^41,42^.
